# Associations between serum ferritin baselines and trajectories and the incidence of metabolic dysfunction-associated steatotic liver disease: a prospective cohort study

**DOI:** 10.1186/s12944-024-02129-6

**Published:** 2024-05-17

**Authors:** Ziping Song, Xinlei Miao, Xiaoling Xie, Guimin Tang, Jiayi Deng, Manling Hu, Shuang Liu, Song Leng

**Affiliations:** 1 Health Management Center, The Second Hospital of Dalian Medical University, Dalian, 116023 Liaoning China; 2 Department of Gastroenterology, The Second Hospital of Dalian Medical University, Dalian, 116023 Liaoning China; 3 School of Public Health, Dalian Medical University, Dalian, 116000 Liaoning China

**Keywords:** Serum ferritin, Metabolic dysfunction-associated steatotic liver disease, Risk factor, Group-based trajectory modeling, Restricted cubic spline, Time-dependent receiver operating characteristic

## Abstract

**Background and aims:**

Evidence from prospective cohort studies on the relationship between metabolic dysfunction-associated steatotic liver disease (MASLD) and longitudinal changes in serum ferritin (SF) still limited. This study aimed to investigate the associations of SF baselines and trajectories with new-onset MASLD and to present a MASLD discriminant model.

**Methods:**

A total of 1895 participants who attended health examinations at least three times in a hospital in Dalian City between 2015 and 2022 were included. The main outcome was the incidence of MASLD. The associations between SF baselines and trajectories with the risk of MASLD were analyzed by Cox proportional hazards regression, restricted cubic spline (RCS) analysis and time-dependent receiver operating characteristic (ROC) curve analysis. In addition, a MASLD discrimination model was established using logistic regression analyses.

**Results:**

Among the 1895 participants, 492 developed MASLD during follow-up. Kaplan-Meier analysis indicated that participants in the low-stable trajectory group had a longer MASLD-free time compared with participants in other groups. Compared with those in the low-stable trajectory group, the adjusted hazard ratios (HRs) with 95% confidence intervals (CIs) for the risk of new-onset MASLD in the medium-high, high-stable and high-high trajectory groups were 1.54(1.18-2.00), 1.77(1.35–2.32) and 1.55(1.07–2.26), respectively (*P*_*trend*_ < 0.001). The results were robust in subgroup and sensitivity analyses. Multivariate Cox proportional regression showed that SF was an independent risk factor of MASLD (*HR* = 1.002, *95%CI*: 1.000-1.003, *P* = 0.003). The restricted cubic spline demonstrated a nonlinear relationship between SF and the risk of MASLD. The 8-variable model had high discriminative performance, good accuracy and clinical effectiveness. The ROC curve results showed that AUC was greater than that of the FLI, HSI and ZJU models (all *P* < 0.01).

**Conclusions:**

Not only a higher baseline SF but also SF changing trajectory are significantly associated with risk of new-onset MASLD. SF could be a predictor of the occurrence of MASLD.

**Supplementary Information:**

The online version contains supplementary material available at 10.1186/s12944-024-02129-6.

## Introduction

Non-alcoholic fatty liver disease (NAFLD) is one of the main causes of chronic liver disease, accounting for approximately 1/3 of the number of patients worldwide, and the prevalence of NAFLD varies from region to region [1–3]. Among Asian countries, China has the highest prevalence, incidence and NAFLD-related annual mortality rate [4]. Increasing evidence has shown that NAFLD can affect multiple organs and can not only progress to cirrhosis or hepatocellular carcinoma, but also increase the risk of suffering from other extrahepatic diseases, such as type 2 diabetes mellitus (T2DM), chronic kidney disease (CKD), cardiovascular disease (CVD) and colorectal cancer, causing great harm to human health [5–9]. Therefore, in 2020, a panel of international experts proposed that metabolic dysfunction-associated fatty liver disease (MAFLD) should replace NAFLD [10]. However, in 2023, three large pan-national liver associations together with diverse organizations and patient rights groups proposed renaming NAFLD to metabolic dysfunction-associated steatotic liver disease (MASLD) [11]. The new name better reflects the influence of metabolic risk factors of this liver disease. Moreover, there is evidence that about 99% of NAFLD patients meet the diagnostic criteria for MASLD [12]. Given that MASLD increases the risk of developing multiple diseases, early identification of high-risk individuals with MASLD has important clinical significance.

In recent years, there have been many articles discussing the correlation between serum ferritin (SF) and MASLD. SF is the main iron storage protein that regulates the storage and metabolism of iron in the body and is related to a variety of metabolic diseases, such as T2DM, hypertension, dyslipidemia, MASLD, and metabolic syndrome (MetS) [13–16]. Approximately 30-40% of MASLD patients have elevated levels of SF, which promotes the occurrence of MASLD through mechanisms such as oxidative stress and insulin resistance [17]. In summary, the onset and severity of MASLD are closely related to SF, and SF can be used as a non-invasive diagnostic method for hepatic steatosis, as demonstrated by previous studies [16, 18, 19]. However, evidence from prospective cohort studies regarding the correlation between SF changing trends and the occurrence of MASLD is limited. Therefore, this study aimed to investigate different trajectories of SF by the group-based trajectory model (GBTM), evaluate the correlation between SF baselines and trajectories and the risk of MASLD and provide a basis for the early identification of MASLD.

## Methods

### Study population

The participants for this study were from the Dalian Health Management Cohort (DHMC) (ChiCTR2300073363). The DHMC is conducted at the Second Hospital of Dalian Medical University, and it is a large ongoing prospective cohort study launched in 2014. Participants were required to complete questionnaires, conduct a standardized health examination and perform laboratory tests to collect their biochemical data. A total of 3219 participants who attended at least three annual health examinations between 2015 and 2022 were included in this dynamic cohort study. Participants who were diagnosed with MASLD at their first physical examination (*n* = 1041) or who had incomplete body mass index (BMI), alanine aminotransferase (ALT), aspartate aminotransferase (AST), γ-glutamyl transpeptidase (GGT), serum uric acid (SUA), serum creatinine (Scr), total cholesterol (TC), triglyceride (TG), high-density lipoprotein cholesterol (HDL-C), or low-density lipoprotein cholesterol (LDL-C) data (*n* = 278) were excluded. Moreover, participants who suffered from other liver diseases, such as autoimmune hepatitis, viral hepatitis, cirrhosis or cancer (*n* = 5), were also excluded. In the end, 1895 participants over 18 years old were enrolled: 985 were male, and 910 were female (Fig. S1). The baseline was defined as the date of the first visit. The outcome of this study was the incidence of MASLD. Participants were followed up until the date of MASLD diagnosis or the date of the last visit, whichever came first. To establish the MASLD discriminant model, 10,665 subjects who attended health examinations between January and December 2018 and met the inclusion and exclusion criteria were included (Fig. S2). The study was approved by the ethical review committee of the Second Hospital of Dalian Medical University(grant number:2,022,064). All participants signed written informed consent.

### Data collection and definitions

The demographic characteristics, including sex, age, history of disease and drinking status, were collected via questionnaire. A measuring instrument was used to measure the height and weight of participants wearing light clothing and barefoot. BMI was calculated as weight (kg) divided by height (m^2^). Blood pressure was measured with an Omron electronic blood pressure monitor (HBP-9020, Japan) after a 5-minute rest. Fasting venous blood was collected, and a Roche Cobasc 501 Chemistry analyzer was used to measure TG, LDL-C, HDL-C, TC, AST, ALT, SUA, and Scr levels.

Hypertension was defined as systolic blood pressure (SBP) ≥ 140 mmHg and/or diastolic blood pressure (DBP) ≥ 90 mmHg or the use of anti-hypertensive drugs, or self-reported hypertension [20]. Diabetes mellitus was identified as treatment with hypoglycemic agents or insulin or a history of diabetes or fasting plasma glucose (FPG) ≥ 7 mmol/L or glycosylated hemoglobin ≥ 6.5% [21]. Dyslipidemia was defined as triglycerides ≥ 150 mg/dL and/or total cholesterol ≥ 200 mg/dL and/or LDL-C ≥ 130 mg/dL and/or HDL-C ≤ 40 mg/dL [22].

### Diagnostic criteria for MASLD

MASLD was diagnosed by qualified and experienced ultrasonographers who reported the presence of steatosis, excluding other causes of steatosis or excessive alcoholic consumption (≥ 20 g/d for females and ≥ 30 g/d for males), with at least one of the following five cardiometabolic risk factors: (1) BMI ≥ 23 kg/m^2^ or waist circumference ≥ 90/80 cm in men and women; (2) FPG ≥ 5.6 mmol/L, or hemoglobin A1c ≥ 5.7%, or T2DM or treatment for T2DM; (3) Blood pressure ≥ 130/85 mmHg or specific drug treatment; (4) TG ≥ 1.70 mmol/L or lipid-lowering treatment; (5) HDL-C < 1.0 mmol/L for men and < 1.3 mmol/L for women or lipid-lowering treatment [11].

### Statistical analysis

When a continuous variable conformed to a normal distribution, it was expressed as the mean$\pm$standard deviation, and one-way analysis of variance (ANOVA) was used for inter-group comparisons. If the data did not follow a normal distribution, they were described as medians (interquartile ranges) and compared by the Kruskal-Wallis H test. Categorical variables were presented as frequencies (proportions), and the differences between the four groups were compared by the chi-square test.

Group-based trajectory modeling (GBTM) is a statistical method based on longitudinal observational data that can assign individuals to distinct subgroups that follow similar trajectories. Therefore, the GBTM was used to determine the trajectory of SF [23]. First, the number of trajectories ranging from 2 to 5 (shapes from linear, quadratic and cubic) was tested. Second, a model that met the following criteria was considered the optimal model: (1) lower absolute value for the Bayesian information criterion (BIC), (2) average posterior probability(AvePP) > 0.7 and (3) each trajectory group had no less than 5% subjects of the total population. The incidence density was calculated with person-years. The Kaplan-Meier method was applied to calculate the cumulative incidence of MASLD and the log-rank test was used to compare differences among groups. Cox proportional hazards regression model and time-dependent ROC curve were used to evaluate the associations between SF trajectory groups and the risk of MASLD, which were further stratified by age, sex, BMI, hypertension, T2DM and dyslipidemia. Moreover, the product interaction term of SF and subgroup factors was included in the Cox regression model to evaluate their interaction with MASLD. The dose-response association between SF and the risk of MASLD was investigated by restricted cubic spline (RCS) analysis.

In addition, to verify the stability of the results, this study conducted three sensitivity analyses. For the first sensitivity analysis, the multiple imputation method was used to fill in the missing baseline covariates, and the GBTM was constructed again. Second, participants were categorized by baseline SF quartiles. Finally, baseline SF was considered a continuous variable for analysis.

Using SPSS (version 27.0) to generate random numbers, 10,644 subjects were randomly divided into a training set (7451 subjects) and a validation set (3193 subjects) at a ratio of 7:3. The MASLD model was constructed based on the results of univariate and multivariate logistic regression analyses. The MASLD model was evaluated using three methods. Discrimination was measured by the area under the ROC curve, and the accuracy was verified using calibration plots. Decision curve analysis (DCA) was applied to evaluate the clinical benefit of the MASLD model. In addition, the model constructed in this study was compared with three common non-invasive diagnostic models using the validation set: fatty liver index (FLI) [24], hepatic steatosis index (HSI) [25], and Zhejiang University index (ZJU) [26]. The calculation formulas are provided in the supplementary material. Delong test was used to compare whether the AUC of this model was statistically different from that of the other three models.

All the statistical analyses were performed with R software version 4.2.2, Stata software version 15.1, and SPSS version 27.0, with *P* < 0.05(two-sided test) indicating statistical significance.

## Results

### Baseline characteristics of the SF trajectories

**Fig. 1:**
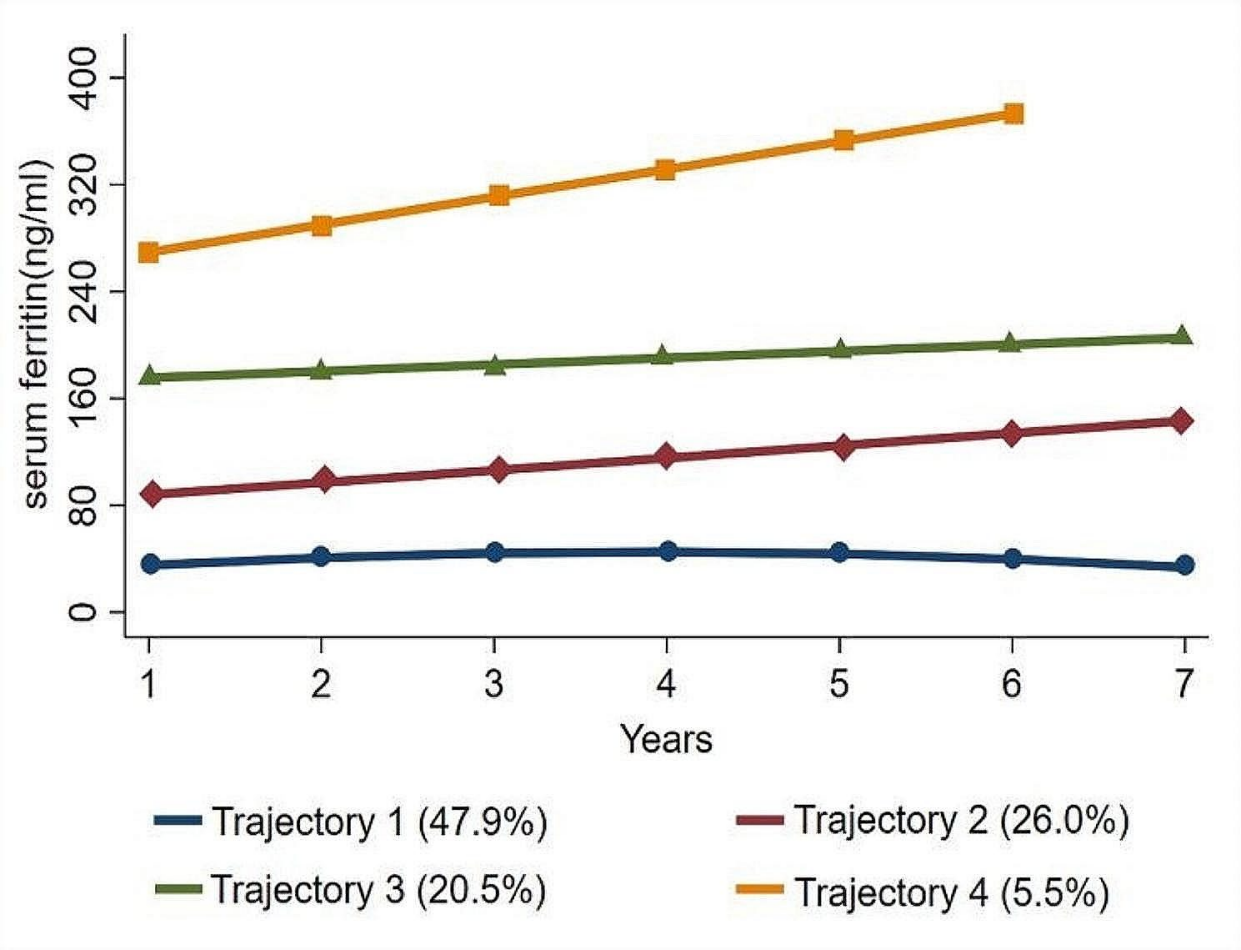
Trajectories of serum ferritin

After multiple fittings of the GBTM, four SF trajectories were identified: a low-stable trajectory (*n* = 923, AvePP = 0.93), a medium-high trajectory (*n* = 478, AvePP = 0.84), a high-stable trajectory (*n* = 390, AvePP = 0.91), and a high-high trajectory (*n* = 104, AvePP = 0.96). As shown in Fig. 1, the SF levels in the low-stable, medium-high, and high-stable trajectory groups remained within the normal range (< 322 ng/mL), while the SF level in the high-high trajectory group gradually increased from the normal range to the abnormal range. The baseline characteristics of the participants stratified by SF trajectories are shown in Table 1. Compared with those in the low-stable trajectory group, the individuals in the medium-high, high-stable and high-high trajectory groups were older, and had higher waist circumference, SBP, DBP, BMI, FPG, TG, TC, LDL-C, SUA, Scr, ALT, AST and GGT, while HDL-C was significantly lower (*P* < 0.001). A total of 492 patients with new-onset MASLD were identified during the follow-up period: 373 were male, 119 were female, and the incidence density of MASLD was 71.85 per 1000 person-years. The incidence density of MASLD in the low-stable, medium-high, high-stable and high-high trajectory groups was 36.99, 94.05, 123.92 and 127.29 per 1000 person-years, respectively.

**Table 1:** Baseline characteristics of the study population according to SF trajectories

Variables	Total (*n* = 1895)	Low-stable (*n* = 923)	Medium-high (*n* = 478)	High-stable (*n* = 390)	High-high (*n* = 104)	*P* value
Gender, n(%)						< 0.001
Male	985 (52.0%)	149 (16.1%)	376 (78.7%)	360 (92.3%)	100 (96.2%)	
Female	910 (48.0%)	774 (83.9%)	102 (21.3%)	30 (7.7%)	4 (3.8%)	
Age, years	41.83 ± 11.84	39.39 ± 11.23	44.90 ± 12.13	43.25 ± 11.34	43.96 ± 13.21	< 0.001
WC, cm	80.95 ± 9.36	76.74 ± 8.74	83.98 ± 8.51	85.39 ± 7.35	87.63 ± 8.27	< 0.001
SBP, mmHg	124.00 ± 15.65	120.39 ± 15.10	127.29 ± 15.36	126.79 ± 15.17	130.38 ± 16.29	< 0.001
DBP, mmHg	74.86 ± 10.80	72.30 ± 10.19	77.32 ± 10.67	77.02 ± 10.73	78.25 ± 11.82	< 0.001
BMI, kg/m^2^	23.22 ± 2.92	22.37 ± 2.88	23.76 ± 2.77	24.20 ± 2.62	24.68 ± 2.68	< 0.001
FPG, mmol/L	5.48 ± 0.87	5.30 ± 0.62	5.63 ± 1.02	5.60 ± 0.83	5.90 ± 1.58	< 0.001
TG, mmol/L	1.25 (0.89, 1.73)	1.13 (0.81, 1.54)	1.28 (0.94, 1.76)	1.42 (1.00, 2.02)	1.50 (1.10, 2.16)	< 0.001
TC, mmol/L	4.83 ± 0.87	4.73 ± 0.83	4.92 ± 0.94	4.92 ± 0.88	4.96 ± 0.79	< 0.001
HDL-C, mmol/L	1.42 ± 0.31	1.51 ± 0.31	1.37 ± 0.32	1.31 ± 0.28	1.29 ± 0.27	< 0.001
LDL-C, mmol/L	2.53 ± 0.66	2.41 ± 0.62	2.63 ± 0.69	2.66 ± 0.66	2.64 ± 0.65	< 0.001
SUA, µmol/L	330.45 ± 81.94	287.41 ± 63.08	358.69 ± 75.58	380.21 ± 71.92	396.02 ± 88.55	< 0.001
Scr, µmol/L	67.19 ± 14.13	59.28 ± 11.58	73.20 ± 12.25	76.47 ± 11.93	75.00 ± 11.12	< 0.001
ALT, U/L	17.00 (13.00, 23.88)	14.53 (11.00, 19.00)	18.86 (15.00, 26.35)	20.30 (15.53, 28.00)	23.00 (15.62, 30.03)	< 0.001
AST, U/L	19.00 (16.25, 22.73)	17.92 (15.59, 21.00)	20.00 (17.00, 23.92)	20.34 (18.00, 24.07)	20.64 (17.53, 25.00)	< 0.001
GGT, U/L	15.34 (11.00, 23.66)	12.21 (9.42, 16.37)	18.08 (13.67, 27.00)	21.16 (15.17, 32.51)	24.40 (16.37, 35.65)	< 0.001
Hypertension, n(%)	319 (16.8%)	104 (11.3%)	115 (24.1%)	73 (18.7%)	27 (26%)	< 0.001
Diabetes, n(%)	64 (3.4%)	13 (1.4%)	21 (4.4%)	19 (4.9%)	11 (10.6%)	< 0.001
Dyslipidemia, n(%)	914 (48.2%)	363 (39.3%)	256 (53.6%)	227 (58.2%)	68 (65.4%)	< 0.001

The data are presented as the means ± SDs, n (%), or medians (quartile 1, quartile 3)

Abbreviations: SF serum ferritin, WC waist circumference, SBP systolic blood pressure, DBP diastolic blood pressure, BMI body mass index, FPG fasting plasma glucose, TG triglyceride, TC total cholesterol, HDL-C high-density lipoprotein cholesterol, LDL-C low-density lipoprotein cholesterol, SUA serum uric acid, Scr serum creatinine, ALT alanine aminotransferase, AST aspartate aminotransferase, GGT γ-glutamyl transpeptidase

### Associations between SF trajectories and the risk of MASLD

**Fig. 2:**
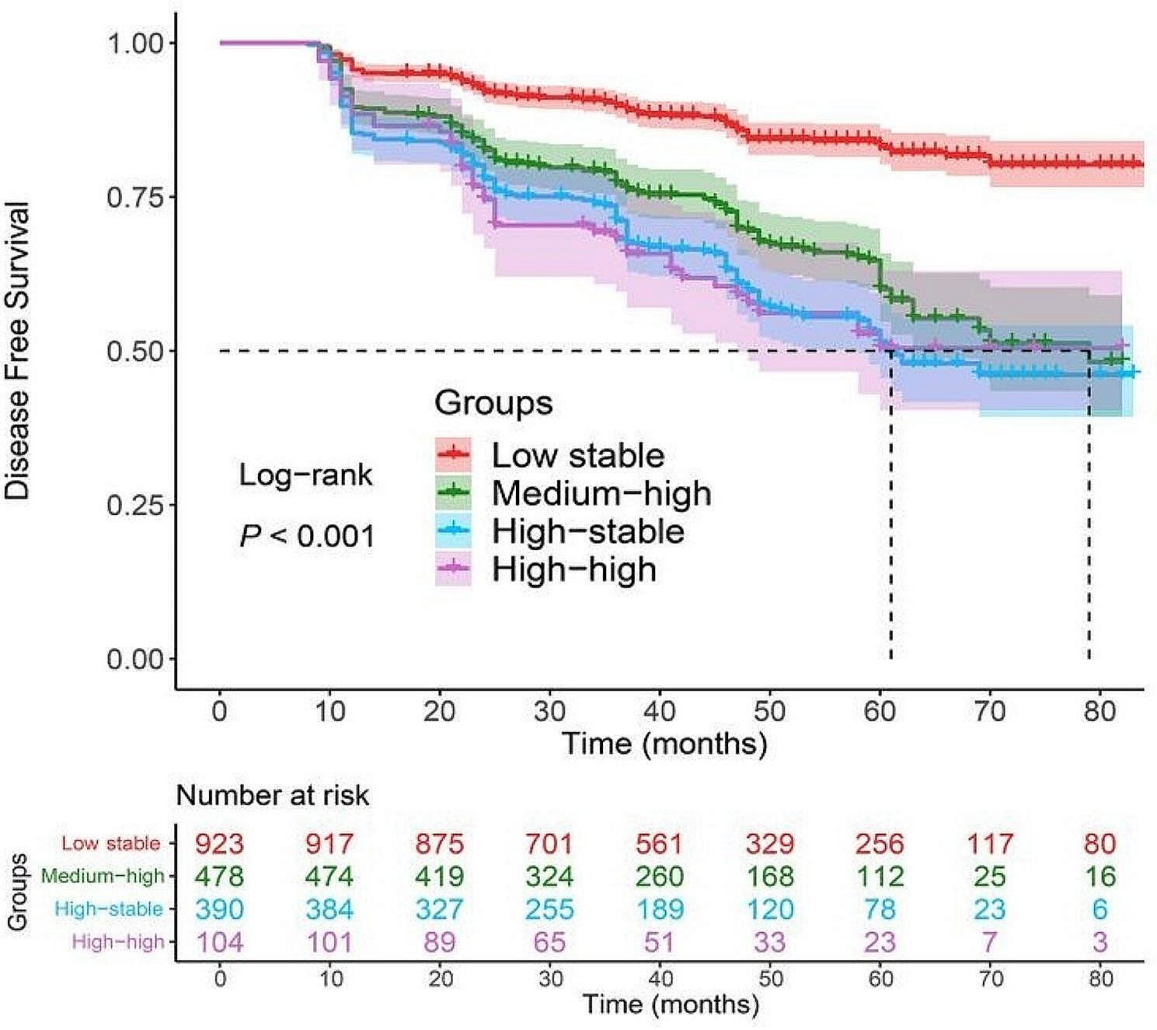
Evaluation of MASLD-free survival in participants with different SF trajectories

**Fig. 3:**
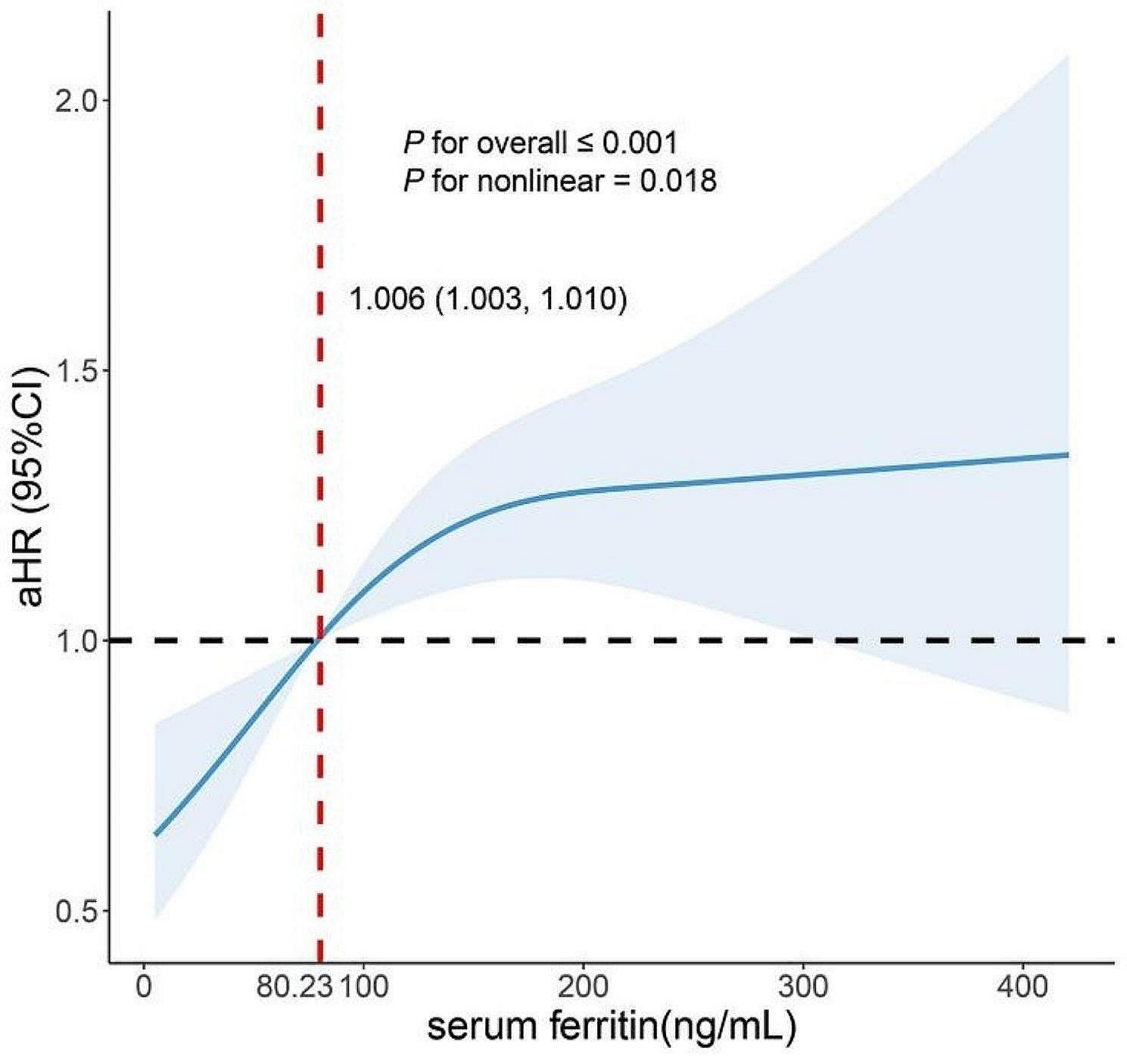
The association between baseline SF and MASLD risk Restricted cubic spline analyses with 3 knots for nonlinear association between baseline SF and MASLD on a continuous scale. *HR*s are indicated by solid lines and 95% *CI*s by shaded areas. Adjusted for age, BMI, SBP, DBP, ALT, AST, GGT, SUA, Scr, TC, TG, HDL-C, LDL-C Abbreviations: HR hazard ratio, CI confidence interval, SF serum ferritin, MASLD metabolic dysfunction-associated steatotic liver disease, BMI body mass index, SBP systolic blood pressure, DBP diastolic blood pressure, ALT alanine aminotransferase, AST aspartate aminotransferase, GGT γ-glutamyl transpeptidase, SUA serum uric acid, Scr serum creatinine, TC total cholesterol, TG triglyceride, HDL-C high-density lipoprotein cholesterol, LDL-C low-density lipoprotein cholesterol

SF trajectories were significantly associated with MASLD. After full adjustment, the HRs of MASLD in the medium-high, high-stable and high-high trajectory groups were 1.54 (*95% CI*: 1.18-2.00, *P* < 0.01), 1.77 (*95% CI*: 1.35–2.32, *P* < 0.01), and 1.55 (*95% CI*: 1.07–2.26, *P* < 0.05), respectively (Table 2). Kaplan-Meier curve analysis revealed that the MASLD-free time of individuals in the medium-high, high-stable and high-high trajectory groups was significantly shorter than that of individuals in the low-stable trajectory group (*P* < 0.001, Fig. 2). In addition, there was a nonlinear relationship between baseline SF and the risk of MASLD after adjusting for other covariates (*P* for nonlinear = 0.018). The risk of MASLD increased when SF concentration was greater than 80.23 ng/ml (*HR* = 1.006, *95% CI*: 1.003–1.010, Fig. 3). The incidence of MASLD in men was approximately 2.90 times greater than that in women (37.9% vs. 13.1%, *P* < 0.001, Table S1). Moreover, with the increase of SF trajectory, the incidence of MASLD was always higher in men than that in women, but there was no significant difference in the high-high trajectory group (Table S1). In the 18 to 44-year-old age group, the incidence of MASLD was lower than that in the 45 and above (21.1% vs. 32.8%, *P* < 0.001, Table S1). The same trend was observed in the low-stable and high-stable trajectory groups, but showed no statistically significant difference in the medium-high and high-high trajectory groups (Table S1). Subgroup analyses were further performed and the interaction test revealed that sex, age, BMI, hypertension status, T2DM status and blood lipid status did not affect the associations between SF trajectories and MASLD incidence (all *P* values for interactions > 0.05, Table 3, Fig. S3).

**Table 2:** Associations between SF trajectories and MASLD risk

Models	Low-stable HR (95%CI)	Medium-high HR (95%CI)	High-stable HR (95%CI)	High-high HR (95%CI)	*P*_trend_
Model 1	1.00	2.53 (2.01–3.20)**	3.35 (2.66–4.23)**	3.46 (2.46–4.87)**	< 0.001
Model 2	1.00	1.94 (1.53–2.46)**	2.45 (1.94–3.10)**	2.21 (1.57–3.13)**	< 0.001
Model 3	1.00	1.54 (1.18-2.00)**	1.77 (1.35–2.32)**	1.55 (1.07–2.26)*	< 0.001

Model 1: Unadjusted

Model 2: Adjusted for age and BMI

Model 3: Adjusted for age, BMI, SBP, DBP, ALT, AST, GGT, SUA, Scr, TC, TG, HDL-C, LDL-C

HR hazard ratio, CI confidence interval, SF serum ferritin, MASLD metabolic dysfunction-associated steatotic liver disease, BMI body mass index, SBP systolic blood pressure, DBP diastolic blood pressure, ALT alanine aminotransferase, AST aspartate aminotransferase, GGT γ-glutamyl transpeptidase, SUA serum uric acid, Scr serum creatinine, TC total cholesterol, TG triglyceride, HDL-C high-density lipoprotein cholesterol, LDL-C low-density lipoprotein cholesterol

*P < 0.05, **P < 0.01

**Table 3:** Subgroup analyses for the association between SF trajectories and MASLD risk

Subgroups	Low-stable HR (95%CI)	Medium-high HR (95%CI)	High-stable HR (95%CI)	High-high HR (95%CI)	*P*_trend_	*P*_interaction_
Gender						0.766
Male	1.00	1.46 (1.01–2.11)*	1.68(1.16–2.43)**	1.45(0.92–2.28)	0.054	
Female	1.00	0.93 (0.57–1.52)	/	/	0.781	
Age						0.582
18–44	1.00	1.60 (1.04–2.44)*	1.65 (1.10–2.49)*	/	0.033	
45–59	1.00	1.46 (0.99–2.16)	1.71 (1.12–2.60)*	1.29 (0.70–2.37)	0.089	
≥ 60	1.00	0.95 (0.43–2.08)	1.78 (0.80–3.96)	1.46 (0.50–4.28)	0.195	
BMI						0.894
18.5 ≤ BMI < 23 kg/m^2^	1.00	1.15 (0.67–1.98)	1.47 (0.85–2.54)	/	0.158	
23 ≤ BMI < 25 kg/m^2^	1.00	1.12 (0.66–1.89)	1.27 (0.74–2.19)	1.08 (0.53–2.18)	0.580	
BMI ≥ 25 kg/m^2^	1.00	1.74 (1.19–2.55)**	1.85 (1.26–2.73)**	2.08 (1.24–3.50)**	0.002	
Hypertension						0.267
Yes	1.00	1.62 (0.97–2.70)	1.81 (1.04–3.13)*	2.31 (1.17–4.55)*	0.011	
No	1.00	1.46 (1.08–1.99)*	1.71 (1.24–2.35)**	1.26 (0.79–2.01)	0.032	
T2DM						0.848
Yes	1.00	1.72 (0.31–9.53)	2.13 (0.35–13.04)	0.55 (0.06–5.08)	0.423	
No	1.00	1.52 (1.16–1.98) **	1.72 (1.30–2.28) **	1.62 (1.10–2.39)*	< 0.001	
Dyslipidemia						0.329
Yes	1.00	1.37 (0.98–1.91)	1.57 (1.12–2.21) **	1.48 (0.94–2.32)	0.025	
No	1.00	1.82 (1.20–2.77) **	2.26 (1.47–3.48) **	/	< 0.001	

Model adjusted for age, BMI, SBP, DBP, ALT, AST, GGT, SUA, Scr, TC, TG, HDL-C, and LDL-C. In each subgroup, variables other than subgroup variables were adjusted

HR hazard ratio, CI confidence interval, SF serum ferritin, MASLD metabolic dysfunction-associated steatotic liver disease, BMI body mass index, SBP systolic blood pressure, DBP diastolic blood pressure, ALT alanine aminotransferase, AST aspartate aminotransferase, GGT γ-glutamyl transpeptidase, SUA serum uric acid, Scr serum creatinine, TC total cholesterol, TG triglyceride, HDL-C high-density lipoprotein cholesterol, LDL-C low-density lipoprotein cholesterol

*P < 0.05, **P < 0.01

/: After stratification by gender, age, BMI, and dyslipidemia, the number of people in the corresponding trajectory group was less than 5%, and they were included in the previous trajectory group for analysis

### Time-dependent ROC analysis for the incidence of MASLD

Figure S4a shows the ROC curves of SF for predicting the onset of MASLD at the 1-year, 5-year, 6-year and 7-year follow-ups. The AUCs at 1, 5, 6 and 7 years were 0.673 (*95% CI*: 0.625–0.721), 0.680 (*95% CI*: 0.641–0.719), 0.739 (*95% CI*: 0.688–0.789) and 0.791 (*95% CI*: 0.646–0.936), respectively. Fig. S4b shows the trend of the changes in the area under the curve (AUC) of SF in predicting the incidence of MASLD from 1 to 7 years. The predictive power of SF for the onset of MASLD gradually increased with time, and the AUC was greater than 0.70 after 5 years.

### Sensitivity analyses

In this study, three sensitivity analyses were conducted. First, after supplementing missing covariates via the multiple imputation method and constructing the GBTM again, a total of 2173 participants were included (Table S2; Fig. S5). Fig. S6 provides variables missing at baseline. It can be seen that SF trajectories were consistently associated with new-onset MASLD, and the HRs in the medium-high and high-stable groups were 1.39 (*95% CI*: 1.07–1.80, *P* < 0.05) and 1.71 (*95% CI*: 1.30–2.26, *P* < 0.01), respectively (Table S3). According to second sensitivity analysis, compared to the lowest quartile of SF, the HRs of MASLD in Quartile 2, Quartile 3, and Quartile 4 in model 3 were 1.005 (*95% CI*: 0.712–1.418, *P* = 0.979), 1.566 (*95% CI*: 1.123–2.185, *P* = 0.008) and 1.570 (*95% CI*: 1.118–2.205, *P* = 0.009), respectively. In addition, the highest SF quartile had the highest risk of MASLD in the three different models (all *P* < 0.01, Table S4). Moreover, after adjusting for confounding factors, the risk of developing MASLD increased by 0.2% for every 1 ng/ml increase in SF in the model that considered SF a continuous variable (*HR* = 1.002, *95% CI*: 1.000-1.003, *P* = 0.003, Table S4). All three sensitivity analyses showed that high levels of SF were associated with an increased risk of MASLD, which is consistent with our previous findings.

### Establishment of the MASLD discriminant model

**Fig. 4:**
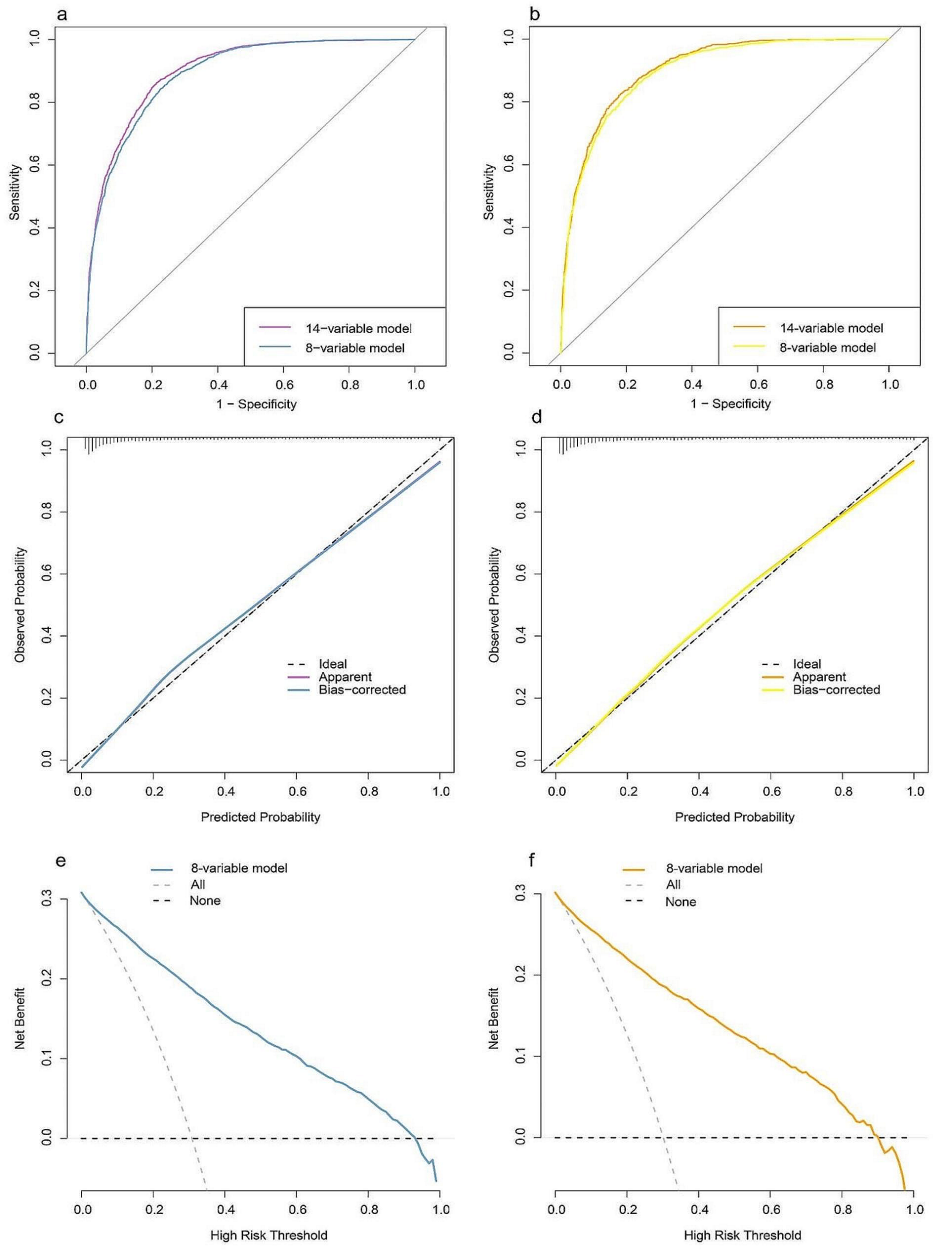
Evaluation of the MASLD model. Analyses of the ROC curves of the 14-variable and 8-variable models for the training (**a**) and validation sets (**b**). Calibration plots of the 8-variable MASLD model in the training (**c**) and validation sets (**d**). Decision curve analysis of the 8-variable MASLD model in the training (**e**) and validation sets (**f**)

According to the univariate analysis, 19 factors, including age, sex, BMI and SF were found to be associated with MASLD (Table S5). The AUCs of BMI, ALT, GGT, HDL-C, TG, SUA, SF, Hb and AST in the training set were greater than 0.70 (Table S6), the optimal cutoff value of SF was 96.280 ng/ml, the AUC was 0.72, the sensitivity was 0.72, and the specificity was 0.61. Variables with multicollinearity were excluded, and less important variables were excluded by the forward: likelihood ratio method. BMI, ALT, GGT, HDL-C, TG, SUA, SF, RBC, WBC, AST, FPG, DBP, Scr and age (Nagelkerke’s R-square 56.3%) were included in the 14-variable model. To simplify the model, SF with other statistically significant variables (AUC > 0.70) were combined. Finally, the 8-variable model included BMI, ALT, GGT, HDL-C, TG, SUA, SF and AST (Nagelkerke’s R-square 53.4%) after simplification. There was no significant difference between the AUCs (0.89 vs. 0.90) of the 8 variables and 14 variables in the training set. Therefore, an 8-variable MASLD discriminant model was constructed (Fig. 4a-b, Table S6). The area under the curve (AUC) of the 8-variable model in the validation set was 0.90 (*95% CI*: 0.88–0.91) (Fig. 4b, Table S6), which was similar to that in the training set, indicating that the model had high discriminative performance. The calibration plot showed that the prediction results of the 8-variable MASLD model were consistent with the actual observations (Fig. 4c-d). DCA demonstrated that the model performed well in clinical practice (Fig. 4e-f). The AUC of the 8-variable model (0.90 (0.88–0.91)) was greater than that of ZJU (0.88 (0.87–0.90)), FLI (0.88 (0.87–0.89)) and HSI (0.87 (0.86–0.88)), indicating that the differentiation of the 8-variable models was better (all *P* < 0.01, Table S7).

## Discussion

This study used the GBTM to identify four distinct longitudinal SF trajectories and showed that not only a higher baseline SF but also SF changing trajectory was significantly associated with an increased risk of new-onset MASLD. Additionally, in different subgroups and sensitivity analyses, this relationship was consistent. As far as we know, this study is the first to investigate the relationship between SF trajectories and MASLD in a prospective cohort study. The time-dependent ROC curve indicated that the SF is a predictor of the occurrence of MASLD, and the predictive power gradually increases with time. After 5 years, the AUC was above 0.70. In addition, because the predictive ability of a single SF is limited, an 8-variable MASLD discrimination model was established by combining SF with other conventional physical examination indicators. ROC curves, calibration plots and DCA were used to evaluate the discriminant performance, accuracy and applicability of the model.

Existing studies have shown that SF is closely related to the incidence and severity of MASLD, but these studies included only SF levels at a single time point [16, 18, 27]. A cohort study in South Korea showed that SF levels can predict the new-onset of MASLD in healthy Korean men [16], which is consistent with the findings of this study. Using the NHANES database, Liao Tan et al. revealed that serum iron status indicators including SF, unsaturated iron binding force (UIBC), transferrin saturation, and hemoglobin, were associated with MASLD and advanced liver fibrosis [28]. Manousou et al. reported that SF is a valuable predictor of non-alcoholic steatohepatitis (NASH) and hepatic fibrosis [29]. A US study involving 628 adults with MASLD confirmed by biopsy showed that after adjusting for sex, age, BMI, ALT and T2DM, an SF > 1.5 $\times$ upper limit of normal (ULN) was independently associated with an increased risk of advanced fibrosis in MASLD patients [18]. Jung et al. used the NLFS to define MASLD and FIB-4 to define liver fibrosis and found that after adjusting for confounding factors, for every 10 ng/mL increase in SF, the ORs of MASLD in males and females were 1.05 and 1.10, respectively, and higher SF levels were associated with a greater risk of hepatic fibrosis [30]. The third sensitivity analysis in this study showed that after adjusting for confounding factors, for every 1 ng/mL increase in SF, the risk of MASLD increased by 0.2%. Both baseline SF and SF trajectories were significantly associated with an increased risk of MASLD, even within the normal range. Moreover, the restrictive cubic spline model showed that when SF concentration was greater than 80.23 ng/ml, the risk of MASLD increased. This suggests that in clinical practice, individuals with high levels of SF (even within the normal range) require moderate physical exercise, a healthy diet, and early screening and management of risk factors to prevent the occurrence of MASLD. Hyperferritinemia is common in ordinary people, and if people suffer from MetS/MASLD, there is a 30-40% chance of detecting it, and it is considered a marker of disease severity [17]. The specific mechanism by which SF promotes the occurrence of MASLD is not yet clear, but epidemiological studies found that this may be related to oxidative stress, insulin resistance, abnormal lipid metabolism, DNA and protein damage, and endoplasmic reticulum stress [17, 31–35]. SF reflects iron storage in the body. Iron overload in the body produces hydroxyl radical, hydrogen peroxide and other reactive oxygen species through chemical reactions, causing oxidative stress, lipid peroxidation, as well as malondialdehyde, 4-hydroxylnonenal and other metabolites accumulation, which increases the membrane fragility of the organelle membrane. The intermediates of lipid peroxidation can interact with DNA bases, lysine and histidine, damaging DNA, proteins and cells. At the same time, it can also promote hepatocyte apoptosis by downregulating the nuclear factor-κB and releasing cytochrome C. Furthermore, studies suggest that adipose tissue plays an important role in MASLD pathogenesis, and iron is a modulator of adipose tissue function. Iron overload in adipose tissue can lead to a decrease in adiponectin and leptin, an increase in resistin and the increased lipolysis of adipocytes, and can also induce insulin resistance in adipocytes.

Hsiao et al. demonstrated that SF was associated with MASLD among obese adults [36]. Liao Tan et al. analyzed SF as a continuous variable and found that SF was related to MASLD in women, participants older than 41 years and participants without diabetes [28]. However, Jung et al. confirmed that higher SF levels are associated with MASLD in both males and females [30]. In this study, the relationship between SF trajectory and MASLD was not influenced by age, sex, BMI, hypertension status, diabetes status, and blood lipids.

Previous studies have shown that age is related to the risk of MASLD, and the prevalence of MASLD increases with age [37, 38], which is consistent with the findings of this study. Lonardo et al. reported sex differences in the prevalence and incidence of MASLD; the prevalence of MASLD in men was greater than that in premenopausal women, while the prevalence and incidence of MASLD in postmenopausal women are on the rise [39]. Due to the lack of information on menstrual history, this study only compared the difference in the incidence of MASLD between men and women and found a greater incidence in men than in women. This sex difference may be due to the influence of estrogen. Studies have confirmed that the production of formyl peptide receptor 2 (FPR2) protective against the liver is mediated by estrogen and that males supplemented with external estrogen will produce more FPR2 and are more resistant to MASLD [40]. Low levels of peroxisome proliferator-activated receptor-γcoactivator 1α (PGC1A) in the liver exacerbated the fatty liver disease associated with an unhealthy diet, and PGC1A expression was higher in the livers of female mice [41]. Limin Liu et al. reported that estrogen decreased caused changes in intestinal flora and short chain fatty acids, and lipid metabolism disorders, which promoted the occurrence of MASLD [42]. Based on the gender and age differences in the prevalence of MASLD, in clinical practice, men should be screened for MASLD as early as possible, while women should focus on the screening of postmenopausal women.

It is well known that the gold standard for diagnosing MASLD is liver biopsy, but this method is traumatic and cannot be applied to large-scale screening. Moreover, most of the existing non-invasive models rely on special serological tests, with poor clinical popularization, and are mostly used for the diagnosis of NASH and liver fibrosis. In addition, the current models are mostly based on the diagnostic criteria of NAFLD, the diagnostic efficacy of MASLD is unknown. In this study, an 8-variable model with high accuracy was constructed, and the ROC curves, calibration plots and DCA showed that the model has strong discriminative performance, accuracy and clinical applicability. Moreover, this model outperforms the ZJU, HSI and FLI models, which can be used to predict the risk of MASLD and enrich non-invasive diagnostic methods for MASLD.

### Strengths and limitations

This study had several advantages. Firstly, in this study, not only the level of SF at a single time point but also its trend of chang over time were included to better understand the correlation between SF and the risk of MASLD. Secondly, an 8-variable model based on physical examination data, including SF was established, which was validated to have high discriminant performance and was superior to the ZJU, FLI and HSI models, providing simple, reliable and objective predictions for the early recognition of MASLD. In addition, there were three sensitivity analyses, so the results were robust. These findings can provide new insight for the prevention, indentification and treatment of MASLD and have important clinical application value. However, this study also had several limitations. Firstly, the participants came from a single center, with a limited sample size, and were all Asian, Therefore, the generalization of the results in different racial or ethnic groups may require further validation of the corresponding large samples. Secondly, eating habits, physical exercise and other lifestyles also have an impact on MASLD, but these factors were not included in the control variables, which may have had a certain impact on the results. It is necessary to conduct further studies in the future.

## Conclusions

In conclusion, not only baseline SF but also the longitudinal trajectory of SF is associated with the incidence of MASLD. This correlation is not affected by sex, age, BMI, hypertension, T2DM or dyslipidemia. SF is an independent risk factor and predictor of the occurrence of MASLD, and the detection of SF is convenient and rapid, which is helpful for identifying individuals at high risk of MASLD.

### Electronic supplementary material

Below is the link to the electronic supplementary material.

Supplementary Material 1

## Data Availability

The datasets used and/or analysed during the current study are available from the corresponding author on reasonable request.

## Abbreviations

NAFLD: non-alcoholic fatty liver disease
MASLD: metabolic dysfunction-associated steatotic liver disease
SF: serum ferritin
HR: hazard ratio
CI: confidence interval
ROC: receiver operating characteristic
CKD: chronic kidney disease
T2DM: type 2 diabetes mellitus
CVD: cardiovascular disease
MetS: metabolic syndrome
BMI: body mass index
WC: waist circumference
SBP: systolic blood pressure
DBP: diastolic blood pressure
GGT: γ-glutamyl transpeptidase
AST: aspartate aminotransferase
ALT: alanine aminotransferase
Scr: serum creatinine
SUA: serum uric acid
HDL-C: high-density lipoprotein cholesterol
TC: total cholesterol
TG: triglyceride
LDL-C: low-density lipoprotein cholesterol
FPG: fasting plasma glucose
GBTM: group-based trajectory modeling
RCS: restricted cubic spline
DCA: Decision curve analysis
FLI: fatty liver index
HSI: hepatic steatosis index
ZJU: Zhejiang University index.
