# Supplementary material for: Associations between serum ferritin baselines and trajectories and the incidence of metabolic dysfunction-associated steatotic liver disease: a prospective cohort study
Source: Lipids Health Dis. 2024 May 17;23:141. doi: 10.1186/s12944-024-02129-6 (PMC11100236; doi:10.1186/s12944-024-02129-6)
Supplement: Supplementary file 1 — Supplementary Material 1 [file 12944_2024_2129_MOESM1_ESM.docx]

**Association between serum ferritin baselines and trajectories and the incidence of metabolic dysfunction-associated steatotic liver disease: a prospective cohort study**

Ziping Song^1, 2^, Xinlei Miao^1^, Xiaoling Xie^3^, Guimin Tang^3^, Jiayi Deng^2^, Manling Hu^2^, Shuang Liu^3^, Song Leng^1, 2 *^

1. Health Management Center, The Second Hospital of Dalian Medical University, 116023, Dalian, Liaoning, China;
2. Department of Gastroenterology, The Second Hospital of Dalian Medical University, 116023, Dalian, Liaoning, China;
3. School of Public Health, Dalian Medical University, 116000, Dalian, Liaoning, China;

**Correspondence:**

Song Leng, MD, Ph.D.

Health Management Center,

The Second hospital of Dalian Medical University,

No.467, Zhongshan Road, Dalian, 116023, China.

Phone:+86-0411-68791226

E-mail: [dllengsong@163.com](mailto:dllengsong@163.com)

ORCID: 0000-0001-6692-919

Supplement

Formulas for calculating FLI^[1]^, ZJU^[2]^ and HSI^[3]^ were as follows: FLI = (e ^0.953 × ln TG (mg/dL) + 0.139 × BMI (kg/m2) + 0.718 × ln GGT (U/L) + 0.053 × WC - 15.745)^/(1 +e ^0.953 × ln TG (mg/dL) + 0.139 × BMI (kg/m2) + 0.718 × ln GGT (U/L) + 0.053 × WC - 15.745)^) × 100; ZJU = BMI (kg/m^2^) + FPG (mmol/L) + TG (mmol/L) + 3 × [ALT (U/L) / AST (U/L)] (+ 2, if female); HSI = 8 × [ALT (U/L) / AST (U/L)] + BMI (kg/m^2^) (+ 2, if diabetes; + 2, if female).

References:

[1] Bedogni G, Bellentani S, Miglioli L, et al. The Fatty Liver Index: a simple and accurate predictor of hepatic steatosis in the general population [J]. BMC Gastroenterol, 2006, 6: 33.

[2] Wang J, Xu C, Xun Y, et al. ZJU index: a novel model for predicting nonalcoholic fatty liver disease in a Chinese population [J]. Sci Rep, 2015, 5: 16494.

[3] Lee J H, Kim D, Kim H J, et al. Hepatic steatosis index: a simple screening tool reflecting nonalcoholic fatty liver disease [J]. Dig Liver Dis, 2010, 42(7): 503-508.

**Additional figures**

**Fig. S1** Flow chart of participants selection

**Fig. S2** Selection of participants for establishing MASLD model

**Fig. S3** Subgroup analysis of associations between SF trajectories and MASLD in terms of gender, age, BMI, hypertension and T2DM

**Fig. S4** Time-dependent ROC curve for predicting the onset of MASLD (A). Trend of AUC (t) over time for predicting the onset of MASLD (B)

**Fig. S5** Serum ferritin trajectories after filling in the baseline missing covariates by multiple imputation method

**Fig. S6** Summary of missing variables

**Additional tables**

**Table S1.** Number of MASLD cases and incidence in different trajectory groups

**Table S2.** Baseline characteristics of participants after filling in the baseline missing covariates by multiple imputation method according to SF trajectories

**Table S3.** Sensitivity analysis for the association between SF trajectories and MASLD risk

**Table S4.** Sensitivity analysis for the association between baseline SF and MASLD risk

**Table S5.** The results of univariate analysis

**Table S6.** AUCs of different variables and models for MASLD discrimination

**Table S7.** Comparison of 8-variable MASLD model with FLI, HSI and ZJU models

**Table S8.** The shape parameters of SF changing trajectories


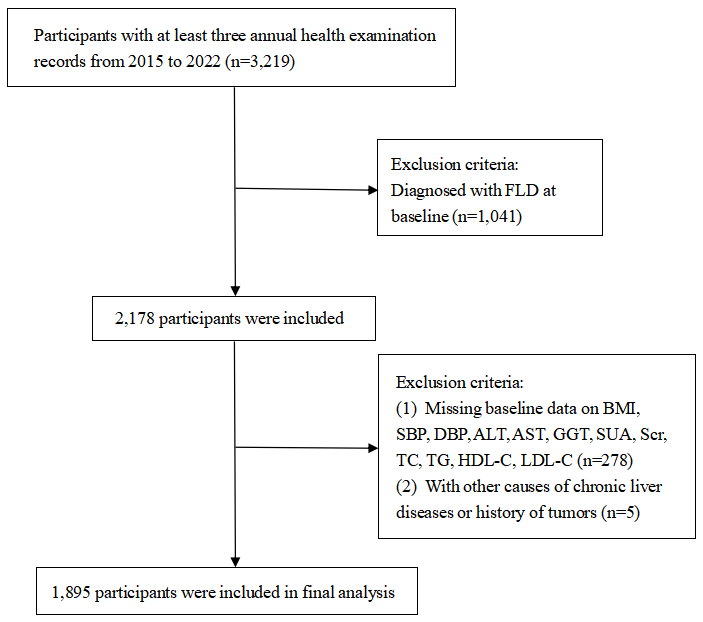


**Fig. S1** Flow chart of participants selection


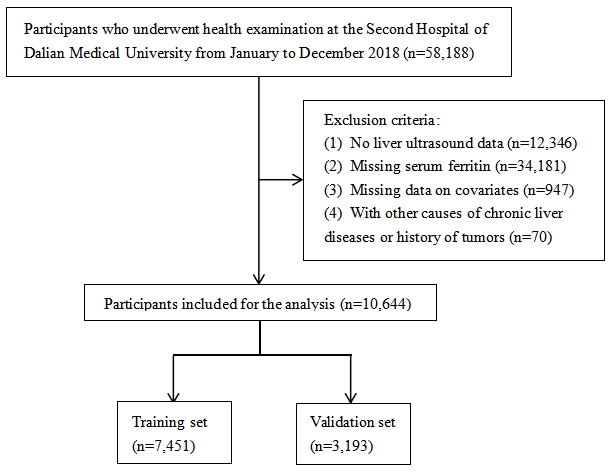


**Fig. S2** Selection of participants for establishing MASLD model

**
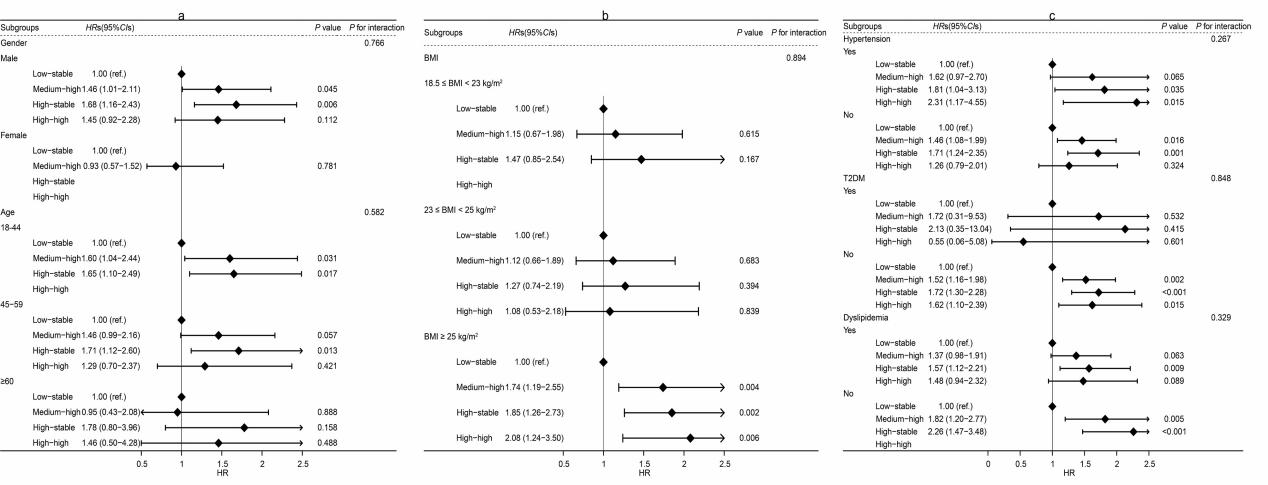
Fig. S3** Subgroup analysis of associations between SF trajectories and MASLD in terms of gender, age, BMI, hypertension and T2DM

Adjusted for age, BMI, SBP, DBP, ALT, AST, GGT, SUA, Scr, TC, TG, HDL-C, LDL-C. Abbreviations: SF serum ferritin, MASLD metabolic dysfunction-associated steatotic liver disease, BMI body mass index, T2DM type 2 diabetes mellitus, SBP systolic blood pressure, DBP diastolic blood pressure, ALT alanine aminotransferase, AST aspartate aminotransferase, GGT γ-glutamyl transpeptidase, SUA serum uric acid, Scr serum creatinine, TC total cholesterol, TG triglyceride, HDL-C high-density lipoprotein cholesterol, LDL-C low-density lipoprotein cholesterol

**
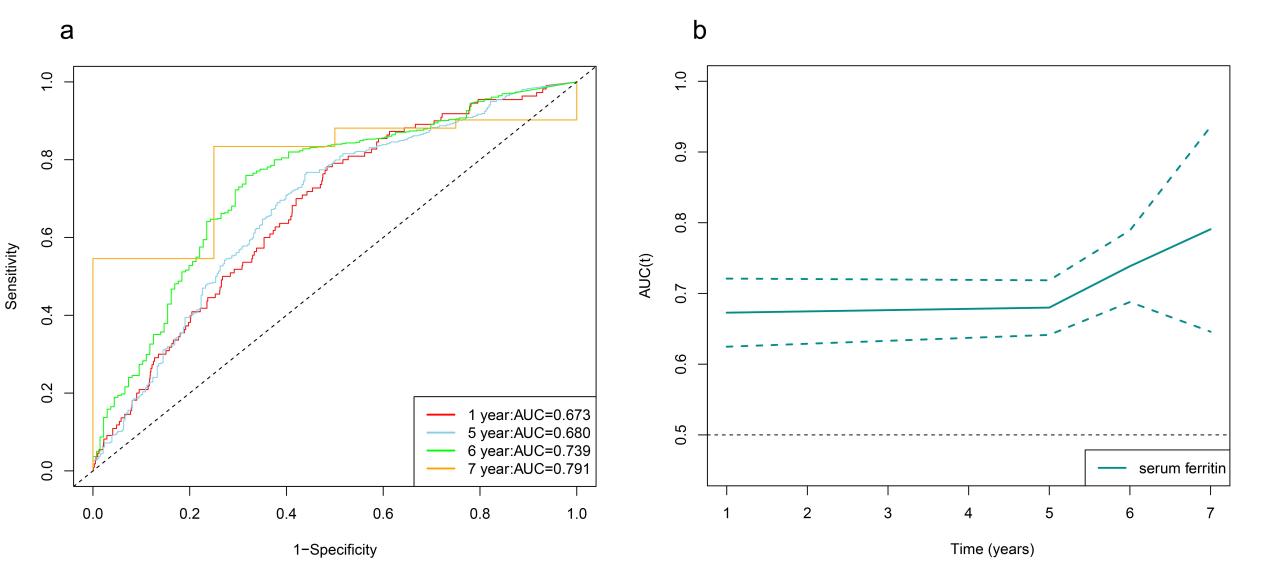
Fig. S4** Time-dependent ROC curve for predicting the onset of MASLD (a). Trend of AUC (t) over time for predicting the onset of MASLD (b)

Abbreviations: ROC receiver operating characteristic curve, MASLD metabolic dysfunction-associated steatotic liver disease, AUC area under the curve


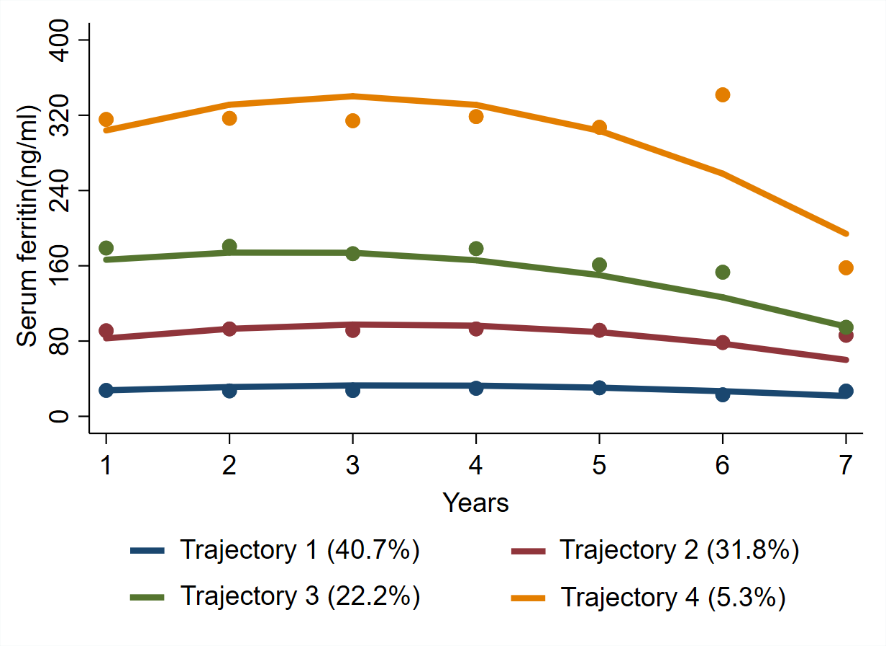


**Fig. S5** Serum ferritin trajectories after filling in the baseline missing covariates by multiple imputation method

A total of 2,173 participants were included. Trajectory 1 (n=897, AvePP=0.92), Trajectory 2 (n=683, AvePP=0.85), Trajectory 3 (n=477, AvePP=0.92), Trajectory 4 (n=116, AvePP=0.97).


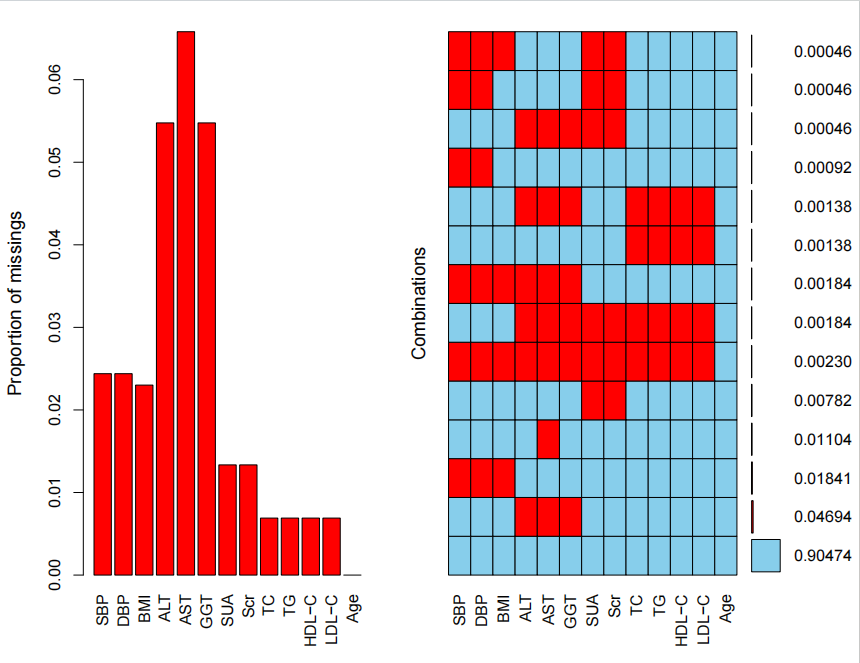
**Fig. S6** Summary of missing variables

Abbreviations: SBP systolic blood pressure, DBP diastolic blood pressure, BMI body mass index, ALT alanine aminotransferase, AST aspartate aminotransferase, GGT γ-glutamyl transpeptidase, SUA serum uric acid, Scr serum creatinine, TC total cholesterol, TG triglyceride, HDL-C high-density lipoprotein cholesterol, LDL-C low-density lipoprotein cholesterol

**Table S1.** Number of MASLD cases and incidence in different trajectory groups

| Variables | Total (n=1895) | Low-stable (n=923) | Medium-high (n=478) | High-stable (n=390) | High-high  (n=104) |
| --- | --- | --- | --- | --- | --- |
| Gender, n(%) |  |  |  |  |  |
| Male | 373/985 (37.9%) | 38/149 (25.5%) | 136/376 (36.2%) | 156/360 (43.3%) | 43/100 (43.0%) |
| Female | 119/910 (13.1%) | 93/774 (12.0%) | 21/102 (20.6%) | 4/30 (13.3%) | 1/4 (25.0%) |
| *P* value | <0.001 | <0.001 | 0.004 | 0.006 | 0.453 |
| Age, n(%) |  |  |  |  |  |
| 18-44 | 233/1106 (21.1%) | 70/624 (11.2%) | 69/227 (30.4%) | 73/203 (40.0%) | 21/52 (40.4%) |
| ≥45 | 259/789 (32.8%) | 61/299 (20.4%) | 88/251 (35.1%) | 87/187 (46.5%) | 23/52 (44.2%) |
| *P* value | <0.001 | <0.001 | 0.252 | 0.039 | 0.877 |

**Table S2.** Baseline characteristics of participants after filling in the baseline missing covariates by multiple imputation method according to SF trajectories

| Variables | Total (n=2173) | Low-stable (n=897) | Medium-high (n=683) | High-stable (n=477) | High-high  (n=116) | *P* value |
| --- | --- | --- | --- | --- | --- | --- |
| Gender, n(%) |  |  |  |  |  | <0.001 |
| Male | 1091 (50.2%) | 79 (8.80%) | 463 (67.8%) | 439 (92.0%) | 110 (94.8%) |  |
| Female | 1082 (49.8%) | 818 (91.20%) | 220 (32.2%) | 38 (8.0%) | 6 (5.2%) |  |
| Age, years | 41.77±11.85 | 38.03±10.46 | 45.17±12.19 | 43.47±11.53 | 43.69±13.21 | <0.001 |
| AC, cm | 80.75±9.33 | 75.74±8.18 | 83.07±8.89 | 85.26±7.38 | 87.25±8.53 | <0.001 |
| SBP, mmHg | 122.00 (113.00, 134.00) | 118.00 (110.00, 128.00) | 125.00 (114.50, 136.00) | 126.00 (117.00, 135.00) | 129.00 (117.00, 137.50) | <0.001 |
| DBP, mmHg | 74.89±10.77 | 71.99±9.72 | 76.64±11.11 | 77.12±10.64 | 77.85±11.85 | <0.001 |
| BMI, kg/m^2^ | 22.77±5.82 | 21.76±4.27 | 23.03±5.07 | 23.62±4.26 | 25.49±15.86 | <0.001 |
| FPG, mmol/L | 5.34 (5.07, 5.66) | 5.21 (4.98, 5.47) | 5.44 (5.15, 5.78) | 5.48 (5.20, 5.80) | 5.50 (5.23, 5.86) | <0.001 |
| TG, mmol/L | 1.24 (0.89, 1.73) | 1.10 (0.81, 1.53) | 1.28 (0.92, 1.75) | 1.39 (0.99, 1.97) | 1.52 (1.14,2.08) | <0.001 |
| TC, mmol/L | 4.83±0.88 | 4.72±0.83 | 4.90±0.91 | 4.91±0.91 | 4.94±0.79 | <0.001 |
| HDL-C, mmol/L | 1.42±0.32 | 1.52±0.31 | 1.39±0.32 | 1.32±0.28 | 1.29±0.27 | <0.001 |
| LDL-C, mmol/L | 2.52±0.66 | 2.39±0.62 | 2.61±0.67 | 2.63±0.68 | 2.64±0.64 | <0.001 |
| SUA, μmol/L | 328.11±81.77 | 280.43±60.06 | 344.59±74.92 | 378.65±73.15 | 392.01±93.13 | <0.001 |
| Scr, μmol/L | 66.76±14.10 | 57.60±10.23 | 70.78±13.21 | 76.31±12.02 | 74.63±10.67 | <0.001 |
| ALT,U/L | 17.00 (12.96, 24.00) | 14.00 (10.94, 19.00) | 18.40 (14.61, 25.53) | 20.37 (15.20, 28.84) | 23.00 (16.19, 30.16) | <0.001 |
| AST,U/L | 19.00 (16.25, 23.00) | 17.74 (15.28, 21.00) | 20.00 (17.00, 23.46) | 20.38 (17.74, 24.22) | 20.76 (17.82, 25.00) | <0.001 |
| GGT,U/L | 15.61 (11.02, 24.20) | 12.00 (9.12, 16.57) | 17.00 (13.00, 25.00) | 20.96 (15.13, 32.82) | 24.04 (16.34, 32.13) | <0.001 |
| Hypertension, n(%) | 386 (17.8%) | 95 (10.6%) | 162 (23.7%) | 97 (20.3%) | 32 (27.6%) | <0.001 |
| Diabetes, n(%) | 73 (3.4%) | 10 (1.1%) | 27 (4.0%) | 25 (5.2%) | 11 (9.5%) | <0.001 |

Data are presented as mean ± SD, n (%), or median (quartile 1, quartile 3).

Abbreviations: AC Abdominal circumference, SBP systolic blood pressure, DBP diastolic blood pressure, BMI body mass index, FPG fasting plasma glucose, TG triglyceride, TC total cholesterol, HDL-C high-density lipoprotein cholesterol, LDL-C low-density lipoprotein cholesterol, SUA serum uric acid, Scr serum creatinine, ALT alanine aminotransferase, AST aspartate aminotransferase, GGT γ-glutamyl transpeptidase

**Table S3.** Sensitivity analysis for the association between SF trajectories and MASLD risk

| Models | Low-stable  *HR* (95%*CI*) | Medium-high  HR (95%CI) | High-stable  *HR* (95%*CI*) | High-high  *HR* (95%*CI*) | *P* _trend_ |
| --- | --- | --- | --- | --- | --- |
| Model 1 | 1.00 | 2.46 (1.96-3.10)** | 3.61 (2.86-4.55)** | 3.67 (2.63-5.13)** | <0.001 |
| Model 2 | 1.00 | 2.23 (1.76-2.82)** | 3.31 (2.62-4.19)** | 3.04 (2.14-4.32)** | <0.001 |
| Model 3 | 1.00 | 1.39 (1.07-1.80)* | 1.71 (1.30-2.26)** | 1.39 (0.95-2.03) | 0.004 |

Model 1: Unadjusted

Model 2: Adjusted for age and BMI,

Model 3: Adjusted for age, BMI, SBP, DBP, ALT, AST, GGT, SUA, Scr, TC, TG, HDL-C, LDL-C

Abbreviations: HR hazard ratio, CI confidence interval, MASLD metabolic dysfunction-associated steatotic liver disease, BMI body mass index, SBP systolic blood pressure, DBP diastolic blood pressure, ALT alanine aminotransferase, AST aspartate aminotransferase, GGT γ-glutamyl transpeptidase, SUA serum uric acid, Scr serum creatinine, TC total cholesterol, TG triglyceride, HDL-C high-density lipoprotein cholesterol, LDL-C low-density lipoprotein cholesterol

*P<0.05, **P<0.01

Sensitivity analysis was performed by supplementing missing covariate data from the baseline

**Table S4.** Sensitivity analysis for the association between baseline SF and MASLD risk

| SF | Model 1 | *P* value | Model 2 | *P* value | Model 3 | *P* value |
| --- | --- | --- | --- | --- | --- | --- |
| Quartile 1 | Reference |  | Reference |  | Reference |  |
| Quartile 2 | 1.319 (0.948-1.835) | 0.100 | 1.135 (0.814-1.582) | 0.454 | 1.005 (0.712-1.418) | 0.979 |
| Quartile 3 | 2.819 (2.105-3.776) | <0.001 | 2.088 (1.551-2.811) | <0.001 | 1.566 (1.123-2.185) | 0.008 |
| Quartile 4 | 3.522 (2.648-4.684) | <0.001 | 2.334 (1.744-3.124) | <0.001 | 1.570 (1.118-2.205) | 0.009 |
| Per 1 SD | 1.004 (1.004-1.005) | <0.001 | 1.003 (1.002-1.004) | <0.001 | 1.002 (1.000-1.003) | 0.003 |

Model 1: Unadjusted

Model 2: Adjusted for age and BMI,

Model 3: Adjusted for age, BMI, SBP, DBP, ALT, AST, GGT, SUA, Scr, TC, TG, HDL-C, LDL-C

Abbreviations: HR hazard ratio, CI confidence interval, MASLD metabolic dysfunction-associated steatotic liver disease, BMI body mass index, SBP systolic blood pressure, DBP diastolic blood pressure, ALT alanine aminotransferase, AST aspartate aminotransferase, GGT γ-glutamyl transpeptidase, SUA serum uric acid, Scr serum creatinine, TC total cholesterol, TG triglyceride, HDL-C high-density lipoprotein cholesterol, LDL-C low-density lipoprotein cholesterol.

**Table S5.** The results of univariate analysis

| Variables | β | $S\overline{x}$ | wald χ2 | *P* value | *HR* (95% *CI*) |
| --- | --- | --- | --- | --- | --- |
| Male | 1.229 | 0.045 | 742.129 | <0.001 | 3.417 (3.128-3.732) |
| Age | 0.023 | 0.002 | 219.020 | <0.001 | 1.023 (1.020-1.026) |
| BMI | 0.456 | 0.010 | 2148.380 | <0.001 | 1.578 (1.548-1.608) |
| SBP | 0.031 | 0.001 | 605.698 | <0.001 | 1.031 (1.029-1.034) |
| DBP | 0.057 | 0.002 | 764.684 | <0.001 | 1.059 (1.054-1.063) |
| WBC | 0.336 | 0.014 | 539.280 | <0.001 | 1.399 (1.360-1.439) |
| RBC | 1.624 | 0.053 | 936.648 | <0.001 | 5.076 (4.574-5.632) |
| Hb | 0.050 | 0.002 | 950.259 | <0.001 | 1.051 (1.048-1.054) |
| ALT | 0.068 | 0.002 | 1280.496 | <0.001 | 1.070 (1.066-1.074) |
| AST | 0.076 | 0.003 | 600.927 | <0.001 | 1.079 (1.072-1.085) |
| GGT | 0.026 | 0.001 | 557.104 | <0.001 | 1.027 (1.025-1.029) |
| SUA | 0.010 | 0.000 | 1324.457 | <0.001 | 1.010 (1.009-1.010) |
| Scr | 0.022 | 0.001 | 229.071 | <0.001 | 1.022 (1.019-1.025) |
| FPG | 0.439 | 0.022 | 403.457 | <0.001 | 1.551 (1.486-1.618) |
| SF | 0.007 | 0.000 | 1030.826 | <0.001 | 1.007 (1.006-1.007) |
| TC | 0.320 | 0.023 | 200.026 | <0.001 | 1.377 (1.318-1.440) |
| TG | 0.974 | 0.028 | 1193.638 | <0.001 | 2.648 (2.506-2.799) |
| HDL-C | -3.530 | 0.093 | 1428.793 | <0.001 | 0.029 (0.024-0.035) |
| LDL-C | 0.461 | 0.031 | 224.565 | <0.001 | 1.586 (1.493-1.685) |

Abbreviations: HR hazard ratio, CI confidence interval, BMI body mass index, SBP systolic blood pressure, DBP diastolic blood pressure, WBC white blood cell, RBC red blood cell, Hb hemoglobin, ALT alanine aminotransferase, AST aspartate aminotransferase, GGT γ-glutamyl transpeptidase, SUA serum uric acid, Scr serum creatinine, FPG fasting plasma glucose, SF serum ferritin, TC total cholesterol, TG triglyceride, HDL-C high-density lipoprotein cholesterol, LDL-C low-density lipoprotein cholesterol

**Table S6.** AUCs of different variables and models for MASLD discrimination

| Variables | Training set (n=7,451) | | | Validation set (n=3,193) | | |
| --- | --- | --- | --- | --- | --- | --- |
|  | AUC (95%*CI*) | Sensitivity | Specificity | AUC (95%*CI*) | Sensitivity | Specificity |
| 14-Variable model | 0.90 (0.89-0.91) | 0.86 | 0.79 | 0.90 (0.89-0.91) | 0.82 | 0.82 |
| 8-Variable model | 0.89 (0.88-0.90) | 0.84 | 0.77 | 0.90 (0.88-0.91) | 0.86 | 0.76 |
| BMI | 0.84 (0.83-0.85) | 0.82 | 0.70 | 0.85 (0.84-0.86) | 0.88 | 0.66 |
| ALT | 0.80 (0.79-0.81) | 0.70 | 0.76 | 0.80 (0.78-0.81) | 0.81 | 0.64 |
| GGT | 0.78 (0.77-0.79) | 0.79 | 0.65 | 0.79 (0.77-0.80) | 0.82 | 0.63 |
| HDL-C | 0.77 (0.76-0.78) | 0.79 | 0.61 | 0.76 (0.74-0.78) | 0.68 | 0.71 |
| TG | 0.75 (0.74-0.76) | 0.65 | 0.71 | 0.76 (0.74-0.77) | 0.64 | 0.74 |
| SUA | 0.75 (0.74-0.76) | 0.72 | 0.67 | 0.73 (0.71-0.75) | 0.67 | 0.68 |
| SF | 0.72 (0.71-0.73) | 0.72 | 0.61 | 0.71 (0.69-0.73) | 0.65 | 0.68 |
| Hb | 0.71 (0.69-0.72) | 0.73 | 0.60 | 0.70 (0.68-0.72) | 0.72 | 0.57 |
| AST | 0.71 (0.69-0.72) | 0.69 | 0.62 | 0.69 (0.67-0.71) | 0.69 | 0.60 |
| RBC | 0.70 (0.69-0.71) | 0.72 | 0.58 | 0.69 (0.67-0.71) | 0.73 | 0.56 |
| FPG | 0.69 (0.68-0.71) | 0.60 | 0.68 | 0.69 (0.67-0.71) | 0.63 | 0.65 |
| DBP | 0.68 (0.67-0.69) | 0.65 | 0.61 | 0.67 (0.65-0.69) | 0.70 | 0.57 |
| SBP | 0.67 (0.66-0.68) | 0.59 | 0.67 | 0.67 (0.65-0.69) | 0.71 | 0.56 |
| WBC | 0.66 (0.64-0.67) | 0.66 | 0.58 | 0.65 (0.63-0.67) | 0.55 | 0.68 |
| Scr | 0.62 (0.61-0.63) | 0.65 | 0.56 | 0.61 (0.59-0.63) | 0.59 | 0.61 |
| LDL-C | 0.60 (0.59-0.61) | 0.48 | 0.68 | 0.60 (0.58-0.62) | 0.67 | 0.50 |
| Age | 0.60 (0.59-0.61) | 0.59 | 0.57 | 0.60 (0.58-0.62) | 0.72 | 0.43 |
| TC | 0.59 (0.58-0.60) | 0.51 | 0.63 | 0.60 (0.58-0.62) | 0.55 | 0.62 |

Abbreviations: AUC area under the curve, MASLD metabolic dysfunction-associated steatotic liver disease, BMI body mass index, SBP systolic blood pressure, DBP diastolic blood pressure, WBC white blood cell, RBC red blood cell, Hb hemoglobin, ALT alanine aminotransferase, AST aspartate aminotransferase, GGT γ-glutamyl transpeptidase, SUA serum uric acid, Scr serum creatinine, FPG fasting plasma glucose, SF serum ferritin, TC total cholesterol, TG triglyceride, HDL-C high-density lipoprotein cholesterol, LDL-C low-density lipoprotein cholesterol

**Table S7.** Comparison of 8-variable MASLD model with FLI, HSI and ZJU models

| Models | AUC (95% *CI*) | Sensitivity | Specificity | Difference AUC (95% *CI*) | *P* value |
| --- | --- | --- | --- | --- | --- |
| 8-Variable model | 0.896 (0.884-0.907) | 0.859 | 0.765 | / | <0.001 |
| ZJU | 0.884 (0.872-0.896) | 0.815 | 0.791 | / | <0.001 |
| FLI | 0.881 (0.868-0.893) | 0.830 | 0.782 | / | <0.001 |
| HSI | 0.872 (0.859-0.884) | 0.823 | 0.775 | / | <0.001 |
| 8-Variable model vs. ZJU | / | / | / | 0.011 (0.004-0.019) | 0.002 |
| 8-Variable model vs. FLI | / | / | / | 0.015 (0.006-0.023) | 0.001 |
| 8-Variable model vs. HSI | / | / | / | 0.024 (0.016-0.032) | <0.001 |
| ZJU vs. FLI | / | / | / | 0.003 (-0.007-0.014) | 0.529 |
| ZJU vs. HSI | / | / | / | 0.012 (0.005-0.020) | 0.001 |
| FLI vs. HSI | / | / | / | 0.009 (-0.003-0.021) | 0.139 |

Abbreviations: MASLD metabolic dysfunction-associated steatotic liver disease, FLI fatty liver index, HSI hepatic steatosis index, ZJU Zhejiang University index, AUC area under the curve

**Table S8.** The shape parameters of SF changing trajectories

|  | Low-stable | Medium-high | High-stable | High-high |
| --- | --- | --- | --- | --- |
| Intercept | 15.52 | 77.91 | 170.36 | 248.73 |
| Linear slope | 12.46 | 9.33 | 4.98 | 20.70 |
| Quadratic slope | -1.60 |  |  |  |
| Group membership probability | 47.90% | 26.00% | 20.50% | 5.50% |
| Group average posterior probability | 0.93 | 0.84 | 0.91 | 0.96 |
| BIC | -41072.17 | | | |
| AIC | -41036.11 | | | |

Abbreviations: SF serum ferritin, BIC Bayesian information criterion, AIC Akaike information criterion
